# Unusual presentations of meningiomas: A case series

**DOI:** 10.4102/sajr.v30i1.3480

**Published:** 2026-09-09

**Authors:** Tavonga P.C.S. Bayela, Christell Ackermann, Morné D. Coetzee, Suzanne O’Hagan

**Affiliations:** 1 Division of Radiodiagnosis, Faculty of Medicine and Health Sciences, Stellenbosch University, Cape Town, South Africa

**Keywords:** atypical meningioma, intraventricular meningioma, cystic meningioma, perineural spread, radiation-induced meningioma

## Abstract

Meningiomas, the most common primary intracranial tumours (accounting for 36% of central nervous system neoplasms), typically present as well-circumscribed, extra-axial masses with homogeneous enhancement, dural tails and hyperostosis on imaging. However, 15% show atypical features that can mimic gliomas, metastases or abscesses, posing diagnostic challenges. This case series present seven histologically confirmed meningiomas illustrating atypical imaging appearances, identified retrospectively from the institutional teaching archive. The cases include intraventricular, septated cystic, perineural spread, circumscribed low T2 or FLAIR signal, frond-like low T2 or FLAIR signal, peritumoural cystic and radiation-induced meningiomas. All were WHO grade 1, except one radiation-induced grade 2 tumour. Key atypical imaging findings included heterogeneous enhancement, non-suppressing cystic components on FLAIR, brain invasion, absent dural tail and perilesional oedema. Although these features suggested higher-grade behaviour, they did not correlate with the final histological grade. Atypical imaging features in meningiomas may closely mimic more aggressive pathologies and do not reliably predict histological grade. Recognition of these variants is essential for accurate diagnosis and appropriate management.

**Contribution:**

This case series expands the recognised imaging spectrum of meningiomas by demonstrating diverse atypical appearances that closely mimic other intracranial pathologies. Importantly, it highlights the discordance between aggressive imaging features and histological grade, reinforcing that atypical morphology should not be equated with higher-grade disease.

## Introduction

Meningiomas originate from meningothelial cells of the arachnoid layer and represent the most prevalent primary brain tumour in adults, with an incidence of 8.3 per 100 000 population.^1^ They are more common in female patients (female-to-male ratio 2:1) and peak in the sixth to seventh decades of life.^1^ According to the 2021 WHO classification, meningiomas are stratified into 15 histological subtypes and three grades based on mitotic activity, brain invasion and other aggressive features.^2,3^ Grade 1 meningiomas constitute 80% – 90% and are benign, while grade 2 (atypical) and grade 3 (anaplastic) account for 5% – 15% and 1% – 3%, respectively.^2,3^ The risk of recurrence increases with grade, reported as 7% – 25% for grade 1, 29% – 52% for grade 2 and 50% – 94% for grade 3.^4^

Classic imaging hallmarks on MRI include isointensity to grey matter on T1- and T2-weighted sequences (T1W and T2W, respectively), avid homogeneous contrast enhancement and a dural tail sign in 60% – 72% of cases.^5,6,7^ CT often reveals hyperdensity (due to cellularity) and adjacent hyperostosis in 20% – 25%.^7^ However, atypical presentations occur in 10% – 15%, including cystic changes (2% – 4%), intraventricular location (0.5% – 5%), perineural spread (rare), low T2 signal (fibroblastic subtype), peritumoural cysts and radiation-induced forms.^6,7^

Cystic meningiomas are classified by Nauta et al. into four types according to the location of the cyst relative to the tumour and the brain: a centrally located intratumoural cyst (type I), a peripherally located intratumoural cyst (type II), a peritumoural cyst lying within the adjacent brain parenchyma (type III), and a peritumoural cyst at the tumour–brain interface containing trapped cerebrospinal fluid (type IV).^8^ Worthington et al. subsequently added a fifth type, in which the cyst surrounds the tumour nodule with neoplastic cells lining the cyst wall (type V).^9^ Types II and V are associated with higher grades.^8^

Atypical features like heterogeneous enhancement, irregular margins, brain invasion, absent calcification, large size (> 4 cm) and peritumoral oedema (> 50% tumour volume) are linked to grade 2 or 3 meningiomas in 60% – 80% of cases, although specificity is low (50% – 70%).^5,10^ Volumetric growth rate (> 2 cm^3^/year) on serial imaging correlates with higher grade.^10^ Perineural spread, though rare in meningiomas (only ~20 reported cases), is antegrade along cranial nerves, unlike retrograde in head-neck cancers, and is not factored into WHO grading.^11^

Radiation-induced meningiomas (RIMs) develop following cranial irradiation, with a latency of 10–30 years, higher multiplicity (30% – 50%) and atypia (20% – 30%).^12,13^ They often show aggressive imaging (heterogeneous enhancement, oedema), but can be grade 1.^13^

## Ethical considerations

Ethical clearance to conduct this study was obtained from the University of Stellenbosch Health Research Ethics Committee (No. C26/02/004). Individual informed consent was obtained from the selected patients.

## Methods

This case series presents seven atypical meningiomas from our institution, highlighting diagnostic pitfalls and the role of MRI in their differentiation. All patients underwent pre- and post-contrast CT scans of the brain. Six of the seven patients also underwent 1.5T MRI (Siemens MAGNETOM Aera) with T1W, T2W, FLAIR and post-gadolinium sequences. Imaging parameters included a slice thickness of 4 mm, a field of view of 230 mm and an intravenous gadolinium dose of 0.1 mmol/kg. One patient was imaged with CT only (unenhanced and contrast-enhanced phases). Histological confirmation was obtained in all cases.

The seven cases were identified retrospectively from the departmental teaching archive between 2017 and 2026 to illustrate the spectrum of atypical imaging appearances of histologically confirmed meningiomas.

## Case presentations

### Case 1: Intraventricular solid-cystic meningioma

A 49-year-old man presented after two episodes of generalised tonic-clonic seizures, and contrast-enhanced (CE) CT imaging (not shown) revealed a solid-cystic mass in the left temporo-parietal region. MRI was performed for accurate localisation and further characterisation (Figure 1). On MRI, the solid-cystic tumour was centred in the left lateral ventricle trigone with parenchymal extension of the cystic component. The intraventricular solid component was isointense to grey matter on T2W and T1W sequences. The cystic component was hyperintense on T2WI and did not suppress on FLAIR, consistent with proteinaceous content or haemorrhage. The solid portion demonstrated heterogeneous enhancement, while the cystic component showed enhancing walls and septations. The mass infiltrated the adjacent parietal lobe parenchyma with an indistinct tumour–brain interface. It measured 6 cm in maximum dimension and was associated with mass effect and peritumoural oedema.

**FIGURE 1:**
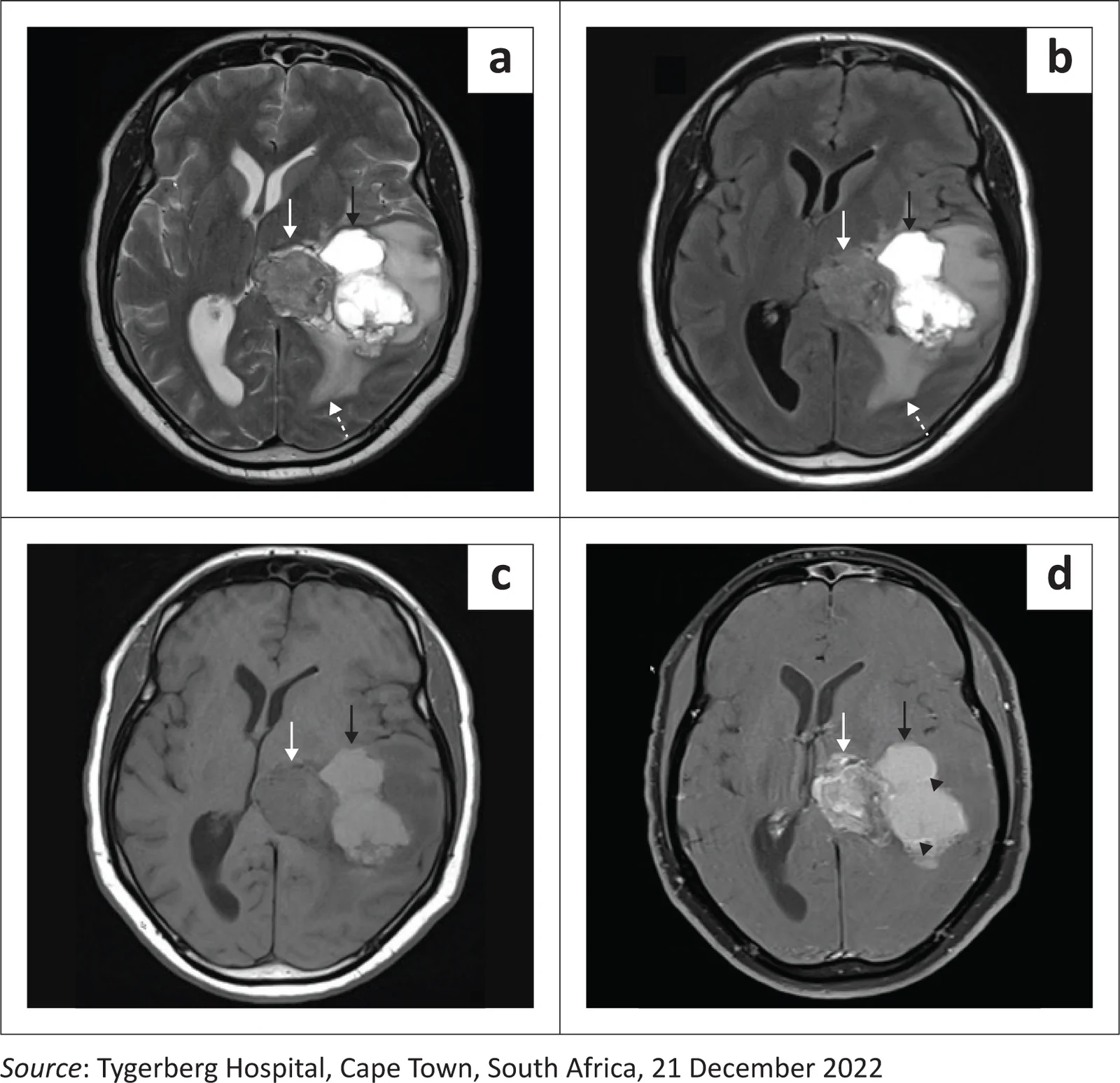
Axial T2-weighted (T2W) (a), axial FLAIR (b) with axial T1-weighted (T1W) pre-contrast (c) and post-contrast (d) MR images demonstrate a solid-cystic mass centred in the trigone of the left lateral ventricle. The solid component (white arrows), located within the body and trigone of the ipsilateral ventricle, is isointense to grey matter on T2W and T1W images and shows heterogeneous post-contrast enhancement. The extraventricular cystic component (black arrows) demonstrates intrinsically high T2W signal without suppression on FLAIR and exhibits peripheral and septal enhancement (arrowheads). T2 hyperintensity is noted in the surrounding white matter (dashed arrows), in keeping with perilesional oedema.

The main differential diagnosis included choroid plexus papilloma and ependymoma. Indistinct tumour–brain interface and extensive peritumoural oedema are associated with brain invasion and with higher grade tumours; however, post-resection histology confirmed a meningothelial meningioma (WHO grade 1).^3^

Intraventricular meningiomas originate from arachnoid cap cells in the choroid plexus. They account for 0.5% – 3% of meningiomas, and most commonly occur in the left atrium (58%).^6,7^ Cystic variants and large tumours with parenchymal invasion can closely mimic intra-axial neoplasms; however, attachment to the choroid plexus and the enhancement pattern are useful diagnostic clues.^6^

### Case 2: Septated cystic meningioma

A 45-year-old woman complained of chronic intermittent headaches and recurrent seizures. Contrast-enhanced CT imaging demonstrated a 5 cm, septated, cystic mass in the left frontal lobe, appearing intra-axial in location (Figure 2). There was surrounding perilesional vasogenic oedema and mass effect. No bone remodelling or dural tail was seen. The cystic morphology, apparent intra-axial location and vasogenic oedema mimicked a cystic glioma. Histology, however, confirmed a microcystic meningioma (WHO grade 1).^3^

**FIGURE 2:**
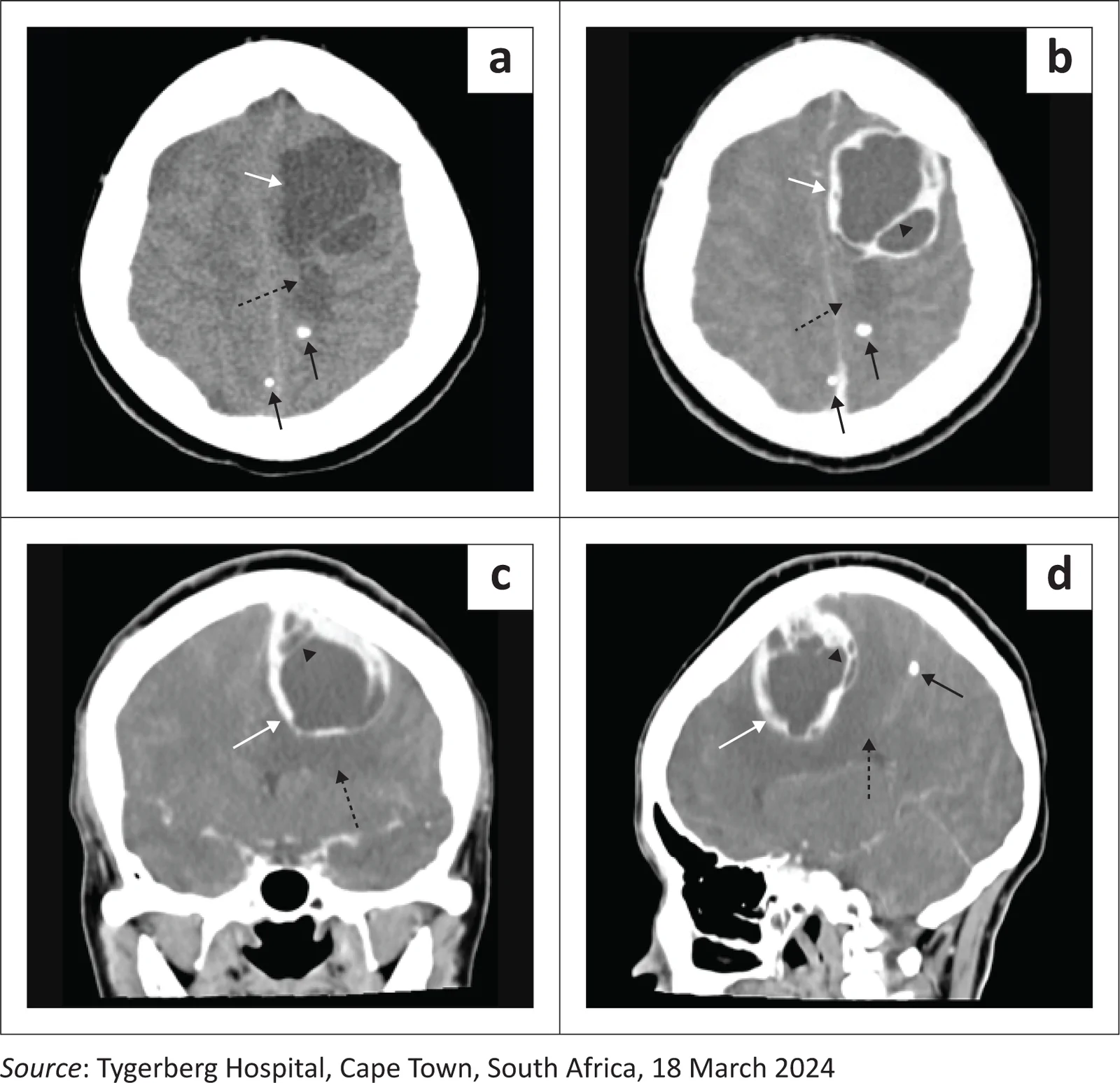
(a, b) Axial pre-contrast (a) and post-contrast (b) CT images, with coronal (c) and sagittal (d) contrast-enhanced (CE) CT reformats, demonstrate a septated, cystic mass in the left frontal lobe (white arrows), appearing intra-axial in location. The post-contrast images show peripheral enhancement and enhancement of the internal septations (arrowheads). There is associated perilesional vasogenic oedema (dashed arrows) and mass effect. No adjacent bone remodelling or dural tail is identified. Incidental calcified granulomas are noted in the bilateral parietal lobes (black arrows).

Microcystic meningioma is a rare histological subtype (1% – 2% of meningiomas) and features vacuolated cells forming multiple cysts, often with a reticular (mesh-like) enhancement pattern.^14^ These tumours are associated with peritumoural oedema in approximately 70% of cases.^14^

### Case 3: Meningioma with perineural spread

A 43-year-old woman presented with chronic left facial pain. Clinical examination revealed left trigeminal (CN V) sensory deficit and ipsilateral otitis media with an effusion. MRI demonstrated a 2.4 cm avidly enhancing extra-axial mass in the left middle cranial fossa (Figure 3). The tumour extended into the cavernous sinus and through the foramen ovale into the masticator space and orbital apex. This accounted for the CN V deficit and provided evidence of perineural spread. Additionally, there was nodular dural thickening in the contralateral middle cranial fossa with associated sphenoid bone hyperostosis, consistent with multiple meningiomas. The contralateral dural-based lesion represented an incidental metachronous meningioma and was managed conservatively.

**FIGURE 3:**
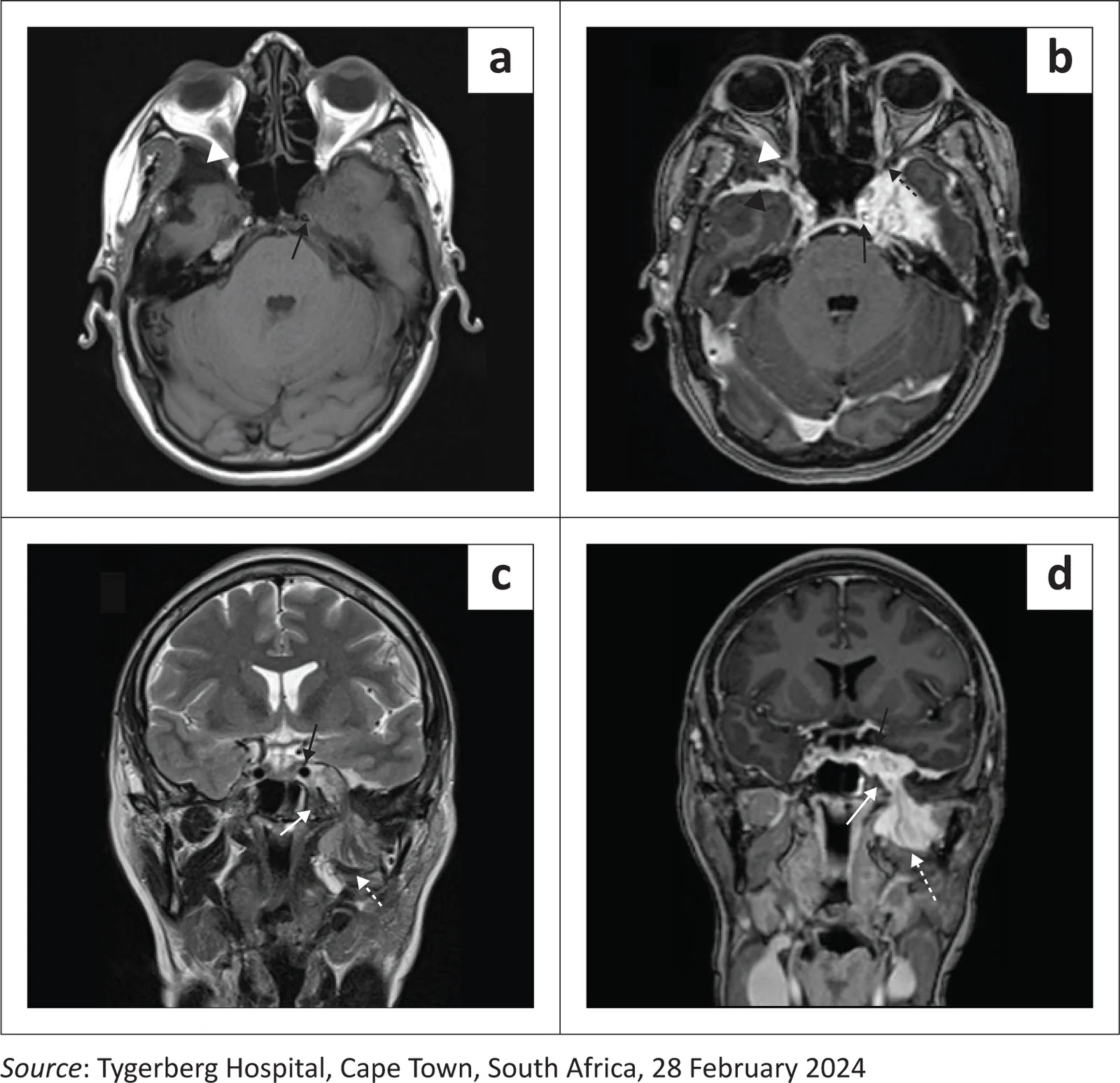
Axial T1-weighted (T1W) pre-contrast (a), 3D axial T1W post-contrast (b), coronal T2-weighted (T2W) (c) and 3D coronal T1W post-contrast (d) MR images demonstrate an avidly enhancing extra-axial mass centred in the left middle cranial fossa. There is perineural spread extending into the cavernous sinus (black arrows) with orbital apex invasion (black dashed arrow) and through the foramen ovale (white arrows) into the masticator space, with pterygoid muscle invasion (white dashed arrows). Additionally, there is nodular dural thickening and enhancement in the right middle cranial fossa (black arrowhead) associated with sphenoid bone hyperostosis (white arrowheads) in keeping with a metachronous meningioma.

Differential considerations were schwannoma, particularly for the left middle cranial fossa mass, and lymphoma. Histology confirmed a meningothelial meningioma (WHO grade 1).^3^

Perineural spread in meningiomas is rare (< 1%) and is typically antegrade, most commonly involving the trigeminal nerve (60% of cases).^11^ Imaging features include nerve thickening and enhancement, as well as foraminal enlargement.^11^

### Case 4: Circumscribed low T2 or FLAIR signal meningioma

A 53-year-old woman presented with acute left-sided hemiparesis. MRI showed a 6.1 cm right frontal convexity extra-axial mass with well-circumscribed margins. The mass was markedly hypointense on T2W and FLAIR sequences with a central focus of hyperintense signal. It had a broad dural attachment without a dural tail and mild perilesional vasogenic oedema (Figure 4). Post-contrast imaging revealed heterogeneous enhancement with a central non-enhancing area corresponding to the T2W hyperintense focus. CT (not shown) confirmed scattered calcifications within this region. No hyperostosis was present at the dural attachment site.

**FIGURE 4:**
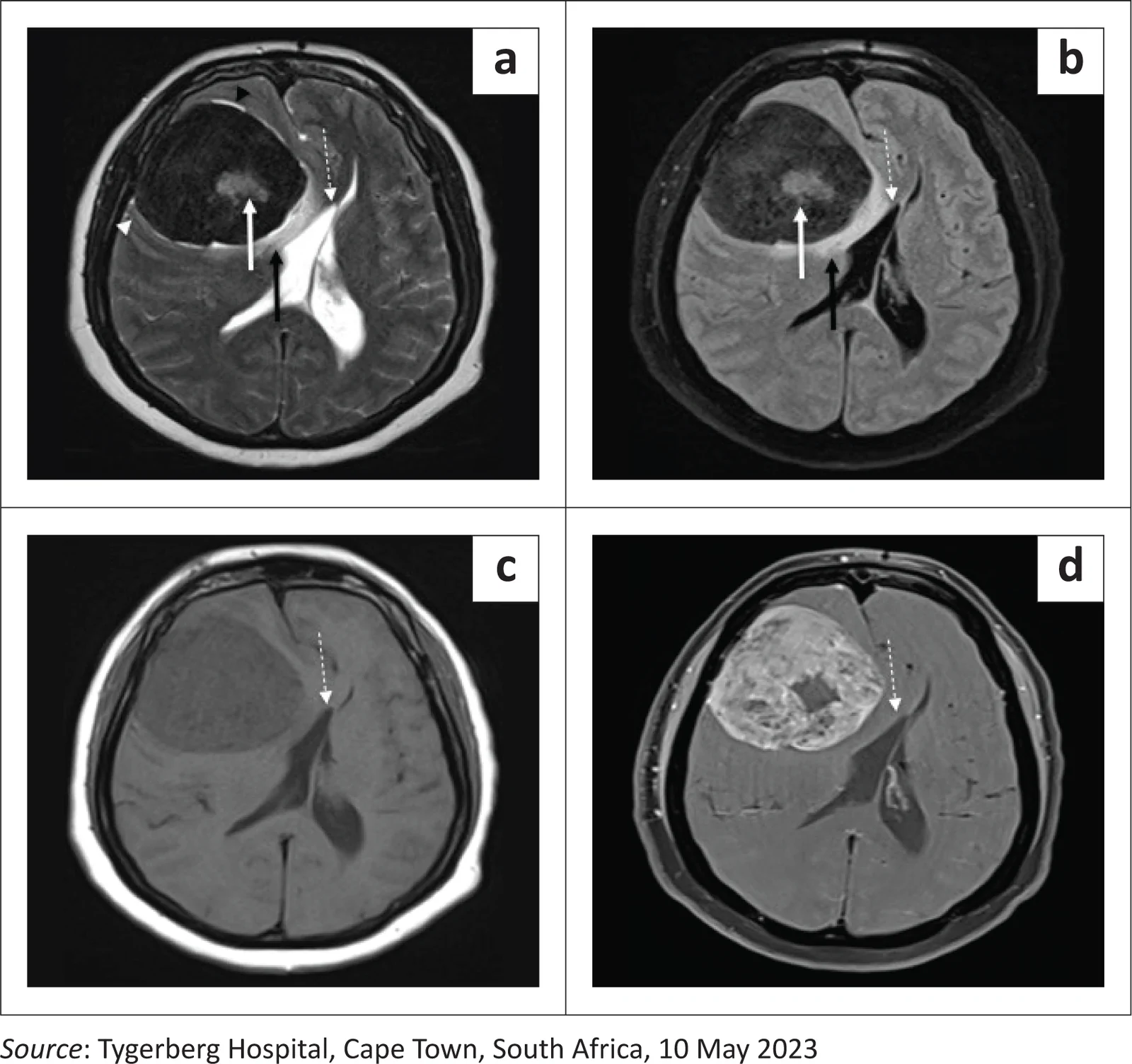
Axial T2-weighted (T2W) (a), axial FLAIR (b), axial T1-weighted (T1W) pre-contrast (c) and post-contrast (d) MR images demonstrate a right frontal extra-axial mass with a CSF cleft (black arrowhead) and buckling of the cortex (white arrowhead), confirming the extra-axial location. The mass is predominantly T2W and FLAIR hypointense with a focal area of marked hyperintense signal (white arrows) resulting in the yin-yang appearance that is typical of solitary fibrous tumour of the dura. The lesion shows heterogeneous post-contrast enhancement. There is associated mild perilesional vasogenic oedema (black arrows) and subfalcine herniation with compression of the frontal horn of the ipsilateral ventricle (dashed arrows).

The primary differential diagnosis was a solitary fibrous tumour of the dura, which classically exhibits very low T2W and FLAIR signal. Histology, however, confirmed a fibroblastic meningioma (WHO grade 1).^3^

Fibroblastic meningiomas account for approximately 10% of meningiomas. They are dense and collagen-rich, resulting in T2W hypointensity in up to 80% of cases.^6^ The combination of marked T2 or FLAIR hypointensity with a central T2-hyperintense non-enhancing focus produced a yin-yang appearance that closely mimicked solitary fibrous tumour of the dura.^15^ Intratumoural calcification, seen in 20% – 30% of fibroblastic meningiomas, further contributes to signal reduction^2^ and favoured a meningioma in this case, as calcification is characteristically absent in solitary fibrous tumours.

### Case 5: Globulated, frond-like meningioma with a low T2-weighted signal

A 29-year-old woman presented with a 7-year history of intractable seizures despite high-dose lamotrigine. CT demonstrated a large, globulated, pre-contrast hyperdense left parietal mass, intralesional focal calcification with heterogeneous post-contrast enhancement and surrounding vasogenic oedema (Figure 5). There was no dural tail or adjacent calvarial hyperostosis. MRI characterised a large, multilobulated, extra-axial left parietal mass with a clear CSF cleft, measuring up to 7.9 cm (Figure 6). The mass was markedly hypointense on T2W and FLAIR images, iso- to hypointense to grey matter on T1W, and showed heterogeneous enhancement on T1W post-gadolinium administration. There was perilesional vasogenic oedema and a mild rightward midline shift.

**FIGURE 5:**
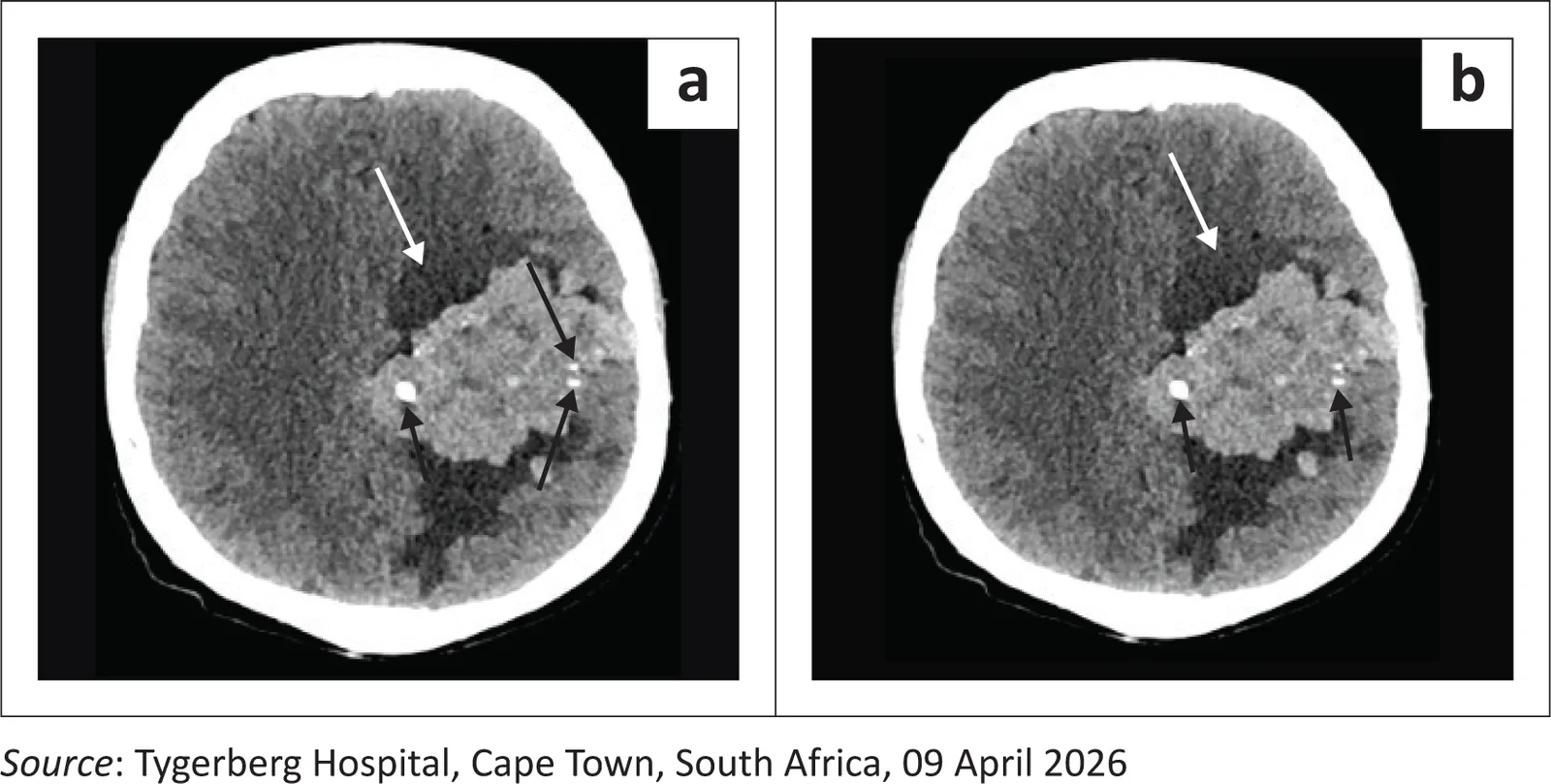
Axial pre-contrast (a) and post-contrast (b) CT images demonstrate a large, globulated, pre-contrast hyperdense left parietal mass with internal calcifications (black arrows), surrounding vasogenic oedema (white arrows) and mild rightward midline shift. No CSF cleft, dural tail or buckling of the cortex visualised on CT.

**FIGURE 6:**
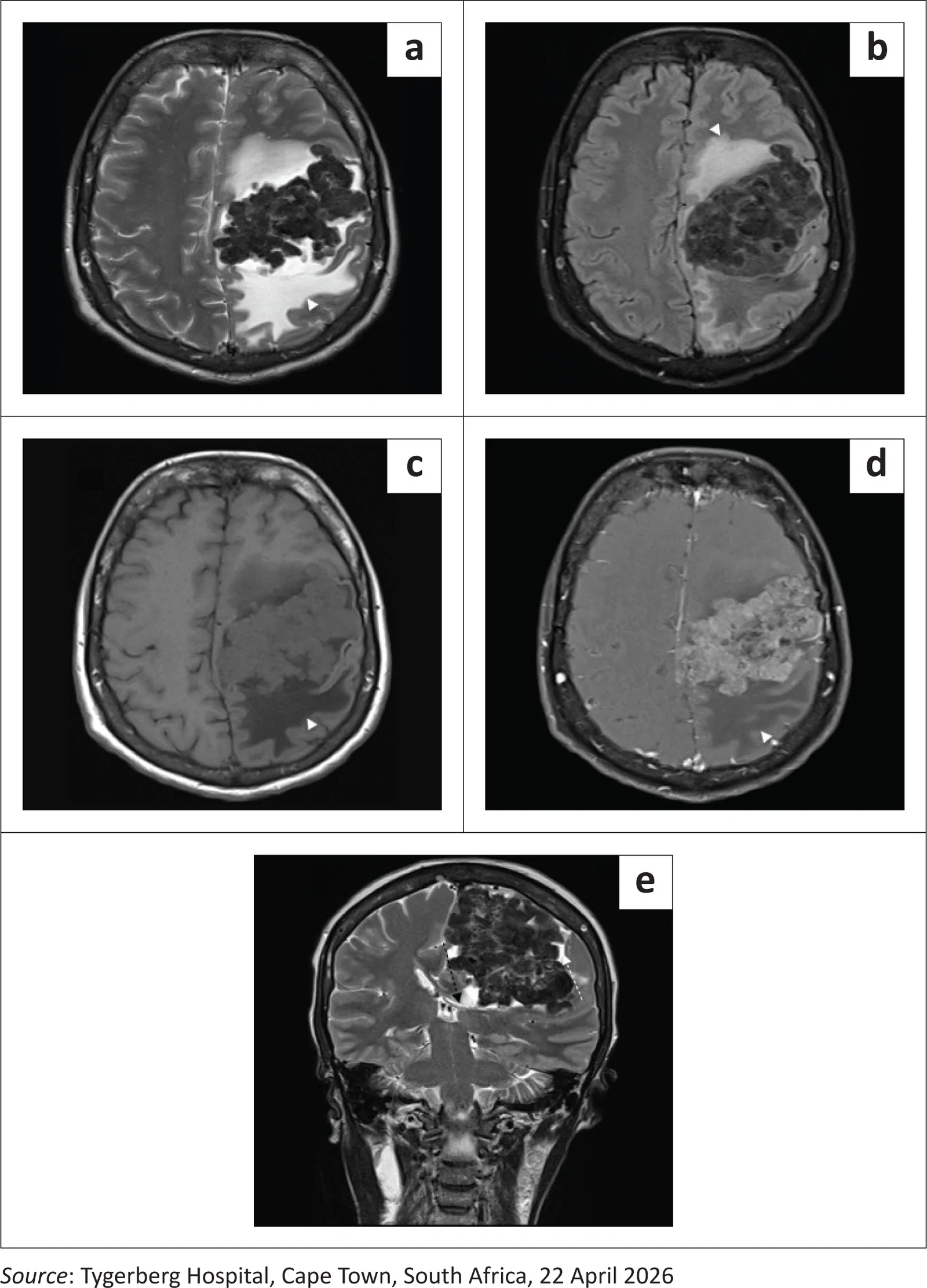
Axial T2-weighted (T2W) (a), axial FLAIR (b), axial T1 pre-contrast (c), axial T1 post-contrast (d) and coronal T2W MR images show a large, multilobulated, extra-axial left parietal mass that is markedly hypointense, with a CSF cleft (white dashed arrow) and surrounding vasogenic oedema (arrowheads). The mass demonstrates heterogeneous enhancement on the post-contrast images. It inferiorly displaces and compresses the left lateral ventricle and temporal lobe (black dashed arrow) with associated rightward midline shift.

The location of the mass could not be ascertained on CT; therefore, differentials for both intra-axial and extra-axial masses were considered, chief among them were oligodendroglioma and solitary fibrous tumour of the dura, respectively. MRI confirmed an extra-axial location, and the marked T2W hypointensity favoured a solitary fibrous tumour of the dura. However, the calcification was atypical.

Histology confirmed a transitional (mixed) meningioma, with features of both the meningothelial and fibrous subtypes (WHO grade 1).^3^ Marked T2W hypointensity in meningiomas reflects dense collagen, fibrous tissue or calcification and is characteristic of the fibroblastic, transitional and psammomatous subtypes.^6,7^ Solitary fibrous tumour of the dura shares this very low T2W signal and avid enhancement, and the two entities cannot be reliably distinguished on imaging alone.^5,6^

### Case 6: Solid mass with peritumoural cyst

A 55-year-old woman complained of chronic headaches. MRI revealed a 6.7 cm left frontal convexity, extra-axial, solid mass with an associated peritumoural cyst. The cystic component showed thin radiating septations and incomplete suppression on FLAIR (Figure 7). There was enhancement of the solid component, cyst walls and septations. CT (not shown) confirmed hyperostosis at the dural attachment site. The mass caused mild mass effect without surrounding vasogenic oedema.

**FIGURE 7:**
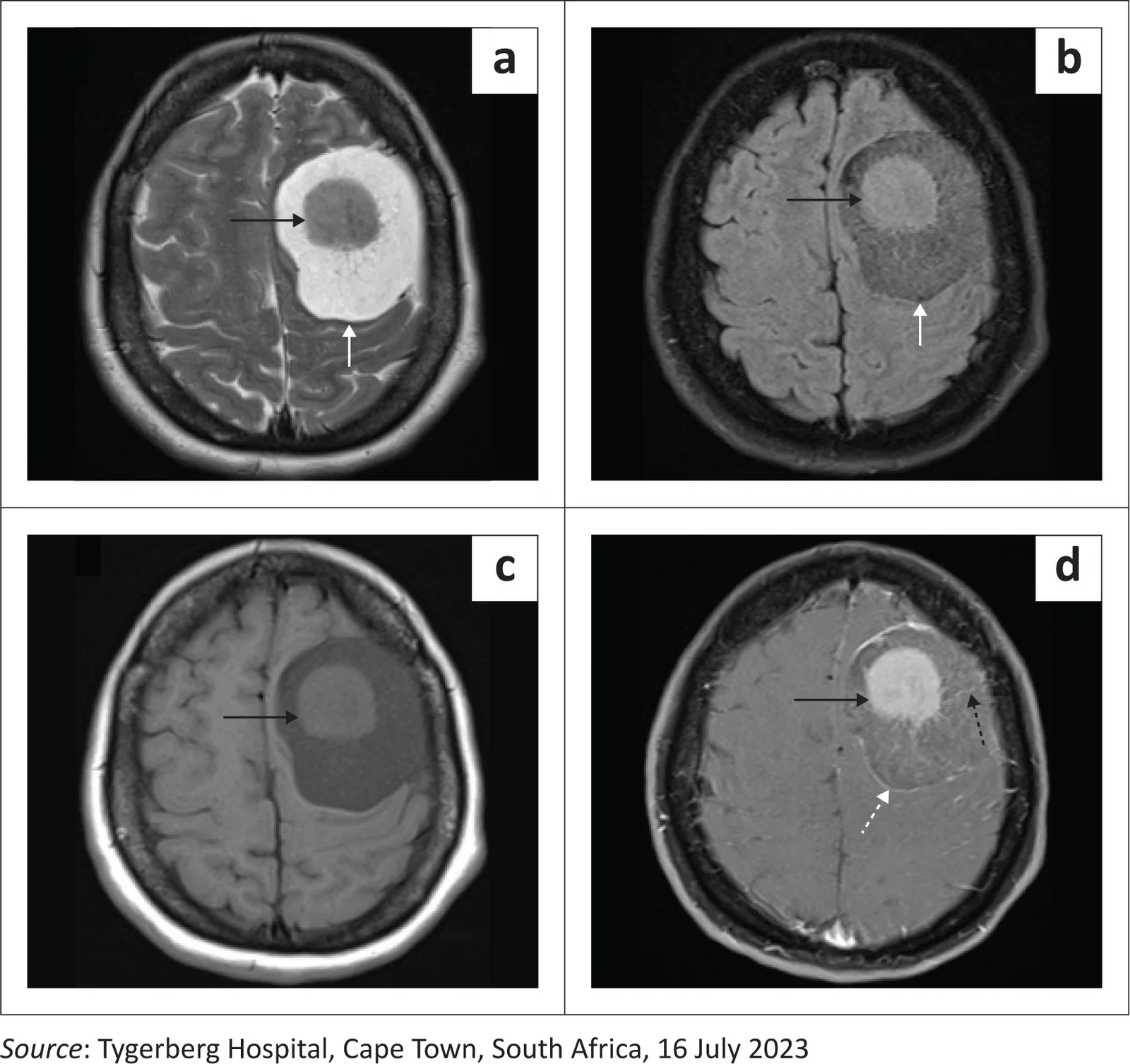
Axial T2-weighted (T2W) (a), axial FLAIR (b), axial T1 pre-contrast (c) and post-contrast (d) MR images demonstrate a left frontal extra-axial mass with an associated peritumoural cyst which does not fully suppress on FLAIR (white arrows). The solid component shows post-contrast enhancement (black arrows), with additional enhancement of the thin radiating septations (black dashed arrow) and cyst wall (white dashed arrow). There is no perilesional vasogenic oedema, but mild mass effect is present.

The imaging appearance resembled a pilocytic astrocytoma; however, the extra-axial location and dural attachment were the decisive features favouring an atypical meningioma. Histology was noteworthy, revealing a meningioma composed of three different cell lines with mixed meningothelial, microcystic and psammomatous features, consistent with WHO grade 1.^3^

Peritumoural cysts (Nauta type 4) are uncommon in meningiomas (4% – 7% of cases). They result from reactive gliosis or trapped cerebrospinal fluid and are most frequently associated with angiomatous subtypes.^8^

### Case 7: Radiation-induced meningioma

A 41-year-old man with a history of left retinoblastoma, treated with radiotherapy and enucleation in childhood, presented with headaches and swelling of the left temporal region. MRI demonstrated a 2.8 cm avidly enhancing mass centred in the left orbit, with extracranial soft tissue invasion and intracranial extension, associated with dural thickening and a dural tail (Figure 8 and Figure 9). There was bone erosion with periosteal reaction seen on CT imaging (Figure 10).

**FIGURE 8:**
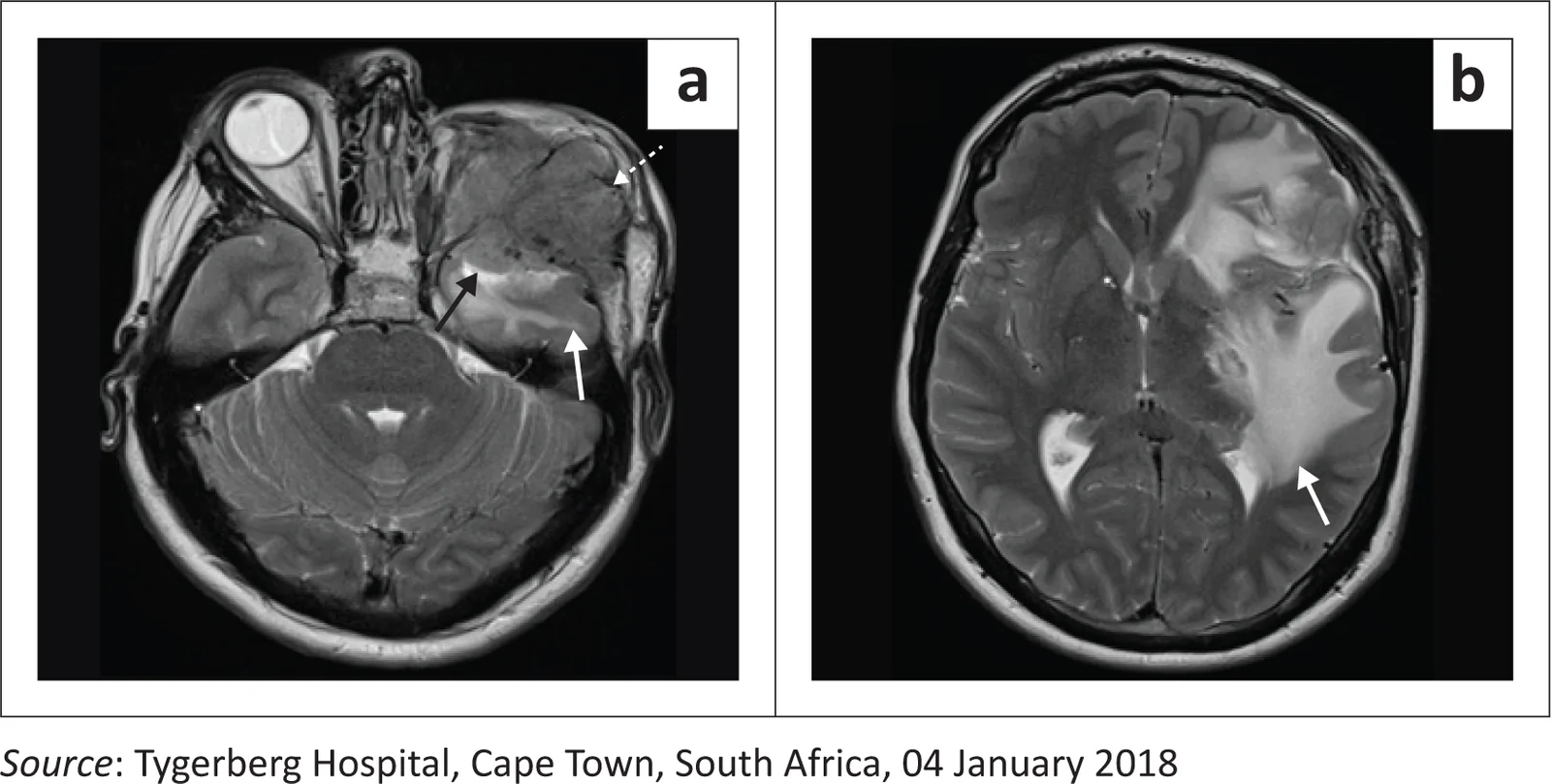
Axial T2-weighted (T2W) MR images at the level of the mid-orbits (a) and lateral ventricles (b) demonstrate a left orbital mass with signal intensity isointense to grey matter, extending into the cranial vault (black arrow) and extracranial soft tissues (dashed arrow). Diffuse T2W hyperintensity is present within the ipsilateral cerebral white matter (white arrows), in keeping with vasogenic oedema.

**FIGURE 9:**
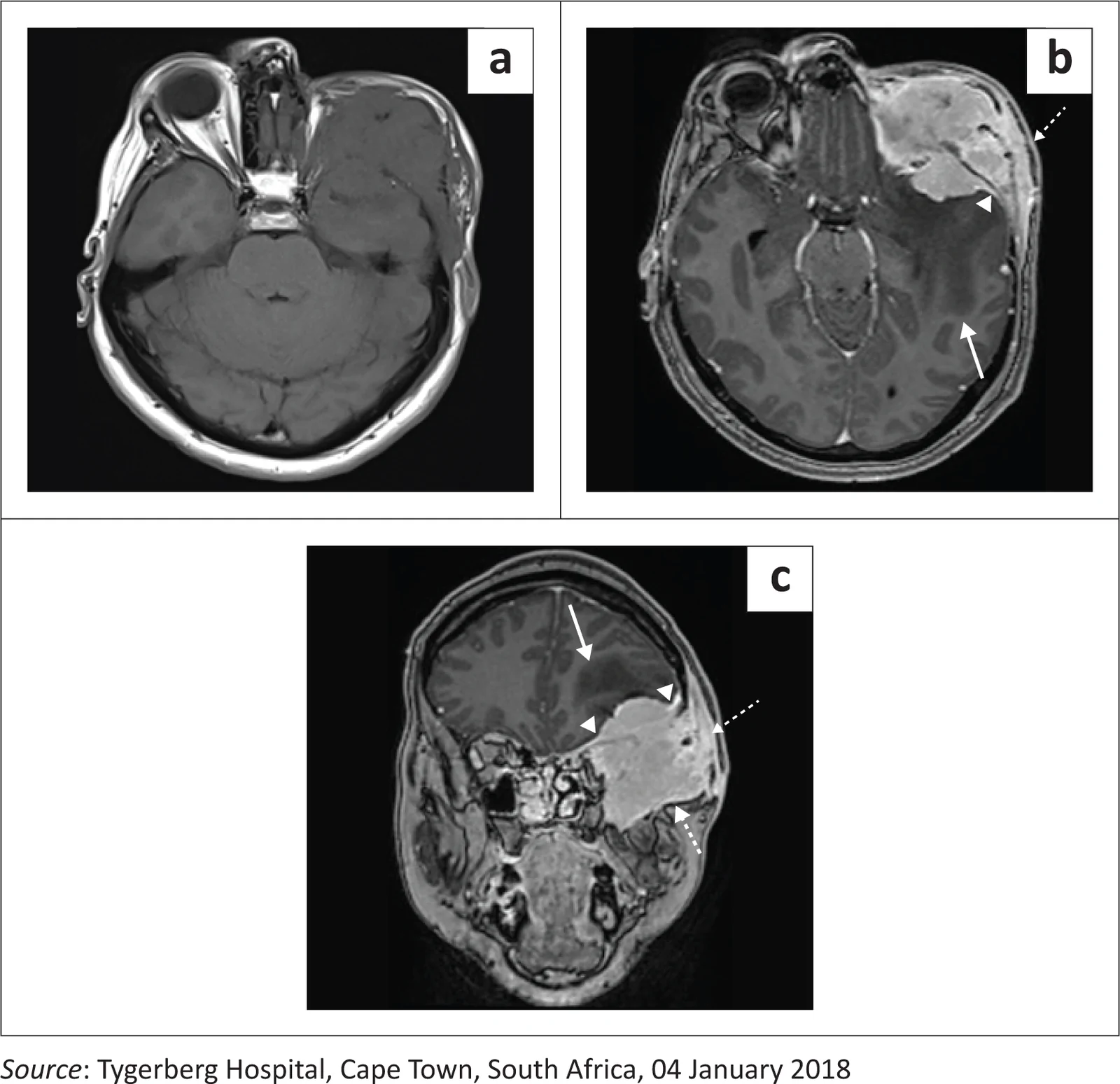
Axial T1-weighted (T1W) pre-contrast (a) and 3D T1W post-contrast axial (b) and coronal MR images demonstrate that the mass is isointense to grey matter on pre-contrast imaging, with intense homogeneous post-contrast enhancement and associated dural thickening and tail (arrowheads). Periorbital soft tissue invasion visualised (dashed arrows). No overt brain invasion despite the diffuse vasogenic oedema (white arrows).

**FIGURE 10:**
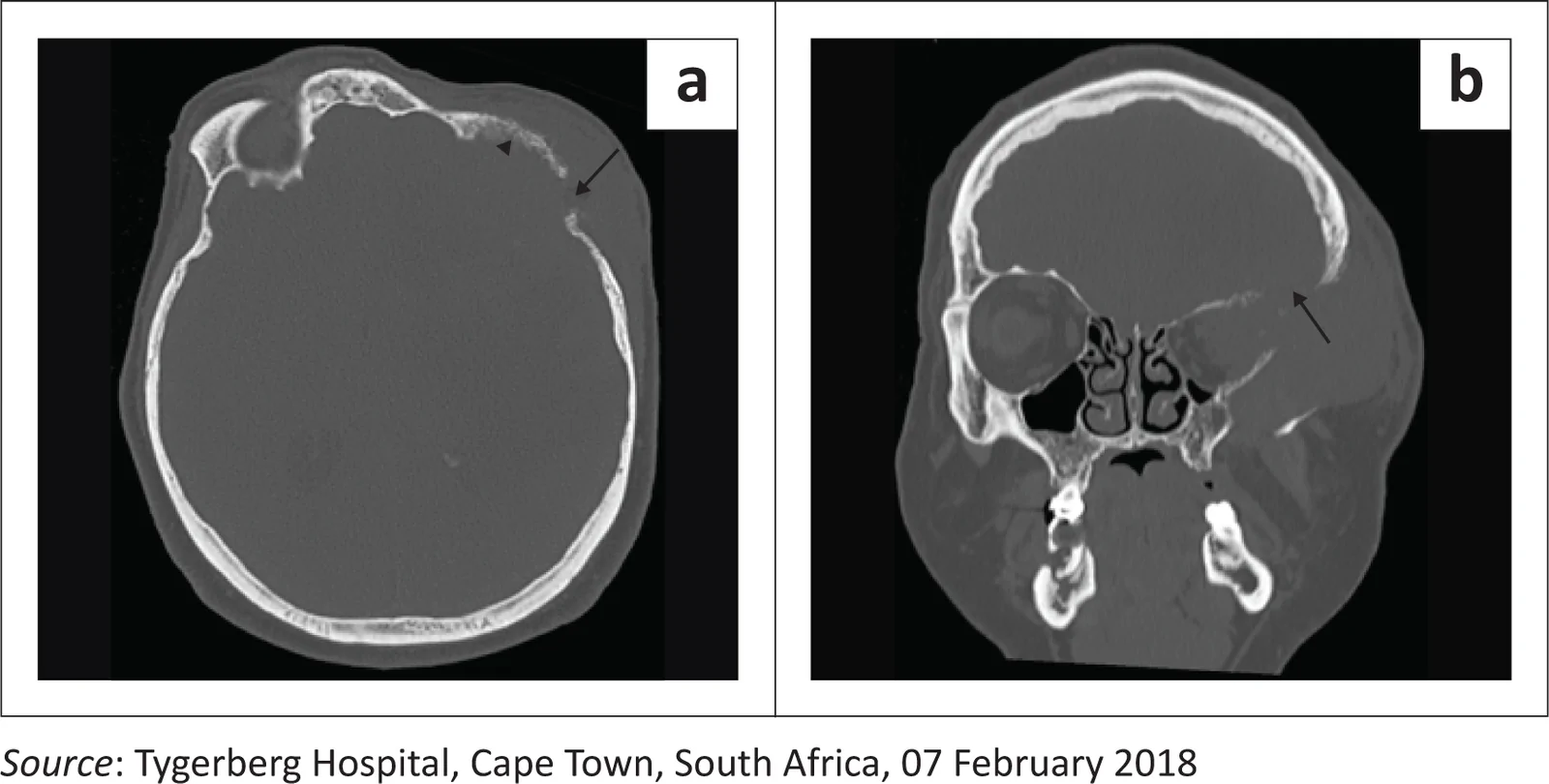
Axial (a) and reformatted coronal (b) CT images in bone window reveal osseous erosion (arrows) with an associated periosteal reaction (arrowhead).

The main concern was a post-radiation rhabdomyosarcoma, due to the multicompartmental extent of the tumour and aggressive bone changes. Histology, however, confirmed a radiation-induced atypical meningothelial meningioma (WHO grade 2).^2,3^ Notably, despite extensive vasogenic oedema, there was no imaging evidence of overt brain invasion, reinforcing the discordance between imaging features and histological grade that is the central theme of this case series. Radiation-induced meningiomas demonstrate atypia in 15% – 20% of cases, have a shorter latency period (typically 10–20 years) and are more often multiple.^12,13^

## Discussion

Atypical meningiomas challenge radiologists due to imaging overlap with malignant entities.^5^ In this series, aggressive features (brain invasion, heterogeneous enhancement, oedema) were present but did not predict tumour grade, aligning with the literature where sensitivity for grade 2 or 3 is 60% – 80%, but specificity is low.^10^ Cystic changes, as seen in cases 1, 2 and 6, occur in 2% – 4%, are often microcystic or secretory subtypes, with non-suppressing cysts indicating neoplasm.^6,8,14^ Perineural spread (case 3) is underreported, potentially due to underdiagnosis; MRI with high-resolution sequences (3D T1 post-contrast) detects nerve enhancement.^11^

Marked T2 hypointensity (cases 4 and 5) reflects dense collagenous or fibrous tissue and/or calcification and characterises the fibroblastic, transitional and psammomatous subtypes, distinguishing them from the typically grey-matter-isointense meningioma.^6,7^ Although these two tumours shared this conspicuous low T2 signal, they differed in both morphology and subtype: the case 4 lesion was circumscribed and fibroblastic, whereas the case 5 lesion was multilobulated and frond-like and proved transitional (mixed meningothelial and fibrous) on histology. This convergence illustrates that marked T2 hypointensity is a non-specific reflection of the shared fibrous substrate rather than a marker of any single subtype, and that neither the signal nor the gross morphology reliably predicts the specific histological subtype. In both cases, the principal imaging mimics solitary fibrous tumour of the dura, which shares a very low T2 signal and avid enhancement and is separable on histology only.^5,6^ Radiation-induced meningiomas (case 7) exhibit atypia, but imaging mimics spontaneous grade 2 meningiomas.^12,13^ Although not obtained in this series, advanced imaging such as perfusion MRI (elevated rCBV in atypicals), spectroscopy (alanine peak) and diffusion (low ADC < 0.8 × 10^{-3} mm^2^/s in high-grade) may improve grading.^5,10^ Volumetric analysis predicts growth.^10^

Two of the seven patients were male (cases 1 and 7). Male sex has been reported as a risk factor for atypical (WHO grade 2) meningioma, although the supporting evidence is moderate rather than definitive.^16^ Notably, in this small series, only case 7 was WHO grade 2, whereas case 1 was WHO grade 1; this association therefore cannot be inferred from these individual cases.^16^ Other risk factors for atypical meningiomas are prior surgery and prior exposure to ionising radiation (case 7).^12,13^

From a practical standpoint, meningioma should remain in the differential of an extra-axial or dural-based mass even when classic signs are absent. Several imaging feature combinations are particularly instructive. A broad dural base with marked T2 and FLAIR hypointensity, with or without calcification, favours a fibroblastic meningioma rather than a higher-grade lesion (cases 4 and 5). A cystic or solid-cystic mass that appears intra-axial but maintains a ventricular or dural attachment, with cyst contents that do not suppress on FLAIR, favours a cystic meningioma over a glioma or ependymoma (cases 1, 2 and 6). Foraminal enlargement with nerve thickening and enhancement along a cranial nerve should raise the possibility of antegrade perineural spread of meningioma (case 3). A new dural-based mass arising within a prior cranial radiation field, even with aggressive imaging features, should prompt consideration of a radiation-induced meningioma (case 7). Because aggressive imaging features do not reliably predict histological grade, the presence of a dural attachment, an extra-axial epicentre or continuity with an arachnoid or ventricular surface remains the most useful anchor for including meningioma in the differential diagnosis.

### Limitations

As a retrospective case series from a single tertiary institution, the small sample size (*n* = 7) carries potential for selection bias towards complex or unusual cases, compounded by the referral bias inherent in a quaternary referral centre. Long-term imaging follow-up was not available for all cases. The descriptive design and small numbers preclude statistically meaningful conclusions regarding correlations between imaging features and histological grade.

Nevertheless, the focused selection of histologically confirmed cases with atypical imaging features provides valuable illustrative examples that highlight diagnostic pitfalls and expand the spectrum of meningioma appearances. The observations are therefore intended to be educational and hypothesis-generating, contributing to greater awareness among radiologists when encountering challenging dural-based lesions.

## Conclusion

While classic imaging features of meningiomas are reliable when present, atypical variants can pose significant diagnostic challenges. Given that meningiomas are the most common primary brain tumour in adults, familiarity with their uncommon and atypical imaging manifestations is essential.^7^ Conventional imaging alone correlates poorly with tumour grade, highlighting the need for additional imaging biomarkers to complement morphological assessment on histology.^2^

This case series illustrates the spectrum of atypical appearances of histologically confirmed meningiomas and emphasises the importance of including meningioma in the differential diagnosis even when imaging features are uncharacteristic.

## References

[1] Ogasawara C, Philbrick BD, Adamson DC. Meningioma: A review of epidemiology, pathology, diagnosis, treatment, and future directions. Biomedicines. 2021; 9(3):319. 10.3390/biomedicines9030319 PMC8004084 33801089

[2] Torp SH, Solheim O, Skjulsvik AJ. The WHO 2021 classification of central nervous system tumours: A practical update on what neurosurgeons need to know – A minireview. Acta Neurochir (Wien). 2022;164(9):2453–2464. 10.1007/s00701-022-05301-y PMC9427889 35879477

[3] Louis DN, Perry A, Wesseling P, et al. The 2021 WHO classification of tumors of the central nervous system: A summary. Neuro Oncol. 2021;23(8):1231–1251. 10.1093/neuonc/noab106 PMC8328013 34185076

[4] Rogers L, Barani I, Chamberlain M, et al. Meningiomas: Knowledge base, treatment outcomes, and uncertainties. A RANO review. J Neurosurg. 2015;122(1):4–23. 10.3171/2014.7.JNS131644 PMC5062955 25343186

[5] Lyndon D, Lansley JA, Evanson J, Krishnan AS. Dural masses: Meningiomas and their mimics. Insights Imaging. 2019;10(1):11. 10.1186/s13244-019-0697-7 PMC6365311 30725238

[6] Kunimatsu A, Kunimatsu N, Kamiya K, Katsura M, Mori H, Ohtomo K. Variants of meningiomas: A review of imaging findings and clinical features. Jpn J Radiol. 2016;34(7):459–469. 10.1007/s11604-016-0550-6 27138052

[7] Buetow MP, Buetow PC, Smirniotopoulos JG. Typical, atypical, and misleading features in meningioma. Radiographics. 1991;11(6):1087–1106. 10.1148/radiographics.11.6.1749851 1749851

[8] Nauta HJ, Tucker WS, Horsey WJ, Bilbao JM, Gonsalves C. Xanthochromic cysts associated with meningioma. J Neurol Neurosurg Psychiatry. 1979;42(6):529–535. 10.1136/jnnp.42.6.529 PMC490257 469560

[9] Worthington C, Caron J, Melanson D, Leblanc R. Meningioma cysts. Neurology. 1985;35(12):1720–1724. 10.1212/wnl.35.12.1720 4069363

[10] Ihwan A, Rafika R, Cangara MH, Sjukur KJ, Faruk M. Correlation between radiological images and histopathological type of meningioma: A cohort study. Ethiop J Health Sci. 2022;32(3):597–604. 10.4314/ejhs.v32i3.16 PMC9214728 35813670

[11] Rastogi S, Bhattacharya K, Mathur A, Sahu AA, Chaudhari A, Shridhar E. Atypical meningioma with perineural spread along hypoglossal nerve. Indian J Radiol Imaging. 2024;34(3):566–568. 10.1055/s-0043-1777743 PMC11188714 38912230

[12] Strojan P, Popović M, Jereb B. Secondary intracranial meningiomas after high-dose cranial irradiation: Report of five cases and review of the literature. Int J Radiat Oncol Biol Phys. 2000;48(1):65–73. 10.1016/S0360-3016(00)00609-X 10924973

[13] Almeida ND, Sinha S, Fang M, et al. Radiation-induced meningiomas: A systematic review and meta-analysis of 927 meningiomas. Surg Neurol Int. 2026;17:35. 10.25259/SNI_1037_2025 PMC12875240 41660311

[14] Kim SH, Kim DG, Kim CY, Choe G, Chang KH, Jung HW. Microcystic meningioma: The characteristic neuroradiologic findings. J Korean Neurosurg Soc. 2003;35: 401–406.

[15] Frazier AA. The Yin and Yang of solitary fibrous tumor. Radiographics. 2014;34(2):294. 10.1148/rg.342144003 24617679

[16] Liang RF, Xiu YJ, Wang X, et al. The potential risk factors for atypical and anaplastic meningiomas: Clinical series of 1,239 cases. Int J Clin Exp Med. 2014;7(12):5696–5700. PMC4307540 25664093

